# A 100-m Sprint Time Is Associated With Deep Trunk Muscle Thickness in Collegiate Male Sprinters

**DOI:** 10.3389/fspor.2019.00032

**Published:** 2019-09-24

**Authors:** Shimpei Fujita, Seiya Kusano, Yusaku Sugiura, Keishoku Sakuraba, Atsushi Kubota, Kazuhiko Sakuma, Yoshio Suzuki, Kohsuke Hayamizu, Yuma Aoki, Masaaki Sugita

**Affiliations:** ^1^College of Health and Welfare, J. F. Oberlin University, Tokyo, Japan; ^2^Company Limited Accel, Tokyo, Japan; ^3^Laboratory of Sports Science, Meikai University, Chiba, Japan; ^4^Graduate School of Health and Sports Science, Juntendo University, Chiba, Japan; ^5^Korea National Track and Field Federation, Chungcheongbuk-do, South Korea; ^6^Laboratory of Food Chemistry, Yokohama University of Pharmacy, Kanagawa, Japan; ^7^Department of Physical Education, Faculty of Sport Science, Nippon Sport Science University, Tokyo, Japan

**Keywords:** multifidus muscle, transversus abdominis, sprint performance, change-point regression model, collegiate athletes

## Abstract

**Introduction:** One reason athletes train their trunk muscles is that the body's trunk stability has been shown to prevent injury. However, the relationship between body trunk muscle thickness, particularly that of deep muscles, and athletic performance remains to be clarified.

**Purpose:** We aimed to explore the relationship between 100-m sprint performance and the sizes of the trunk stabilizing muscles, the psoas major muscle (PM), transversus abdominis (TA), and multifidus muscle (MM), in collegiate sprinters.

**Methods:** Fourteen male sprinters belonging to a university athletics club participated in this study. The thicknesses of the TA and MM were measured using an ultrasonic diagnostic apparatus (ProSound C3; Aloka, Tokyo, Japan). The cross-sectional area of the PM was assessed by a magnetic resonance imaging apparatus (Vantage Elan; Toshiba Medical Systems, Tokyo, Japan). The relationship between these anthropometric parameters and the 100-m sprint time was analyzed by Spearman's correlation coefficient, multi- regression analysis, and the change-point regression model.

**Results:** The sizes (mean ± SD) of the muscles were: PM, 43.074 ± 7.35 cm^2^; TA, 4.36 ± 0.72 mm; and MM, 3.99 ± 0.48 cm. The mean 100-m sprint time was 11.00 ± 0.48 s. Spearman's correlation analysis revealed that the 100-m sprint time had a significant moderate negative correlation with TA (ρ = −0.691, *p* < 0.01) and a low negative but not significant correlation with MM (ρ = −0.327, *p* = 0.28), whereas PM did not show a significant or in-negligible correlation. The change-point regression model found the change-points in the 100-m sprint time and the thickness of the TA and MM at 4.70 mm (95% CI: 4.00–5.43 mm) and 3.84 cm (95% CI: 3.28–4.31 cm), respectively. The sprint time decreased with an increase in the thickness of the muscles up to the change-points, whereas it did not change even if the muscles became thicker than the change-points. The change-points were consistently observed when the thickness of the muscles was normalized by body mass.

**Conclusion:** Sprint performance for 100-m was found to be associated with TA and MM thickness in a biphasic manner. As muscle thickness increased, the sprint time decreased, followed by a plateau phase.

## Introduction

The athletic 100-m sprint is one of the most popular events at the Olympic Games, where leading sprinters from around the world compete. Evaluation of the top 100-m sprinters shows that the key factors affecting 100-m sprinting performance are high sprinting speed and abilities to generate explosive acceleration and maintain high sprinting speed (Majumdar and Robergs, 2011). Additionally, anteroposterior force/power and horizontal force have been reported to be involved in the sprinting speed up to 40 m (Rabita et al., 2015). The factors that generate these forces are classified as controllable and uncontrollable factors: the controllable elements primarily include height, limb length, and cross-sectional muscle area (Rabita et al., 2015).

In recent years, the body trunk has attracted attention in terms of performance improvements and injury prevention (Zattara and Bouisset, 1988; Leetun et al., 2004) Indeed, the muscles located in the body trunk are at the center of all motor chains and are important for the stability of the spine and pelvis (Putnam, 1993). In addition, the trunk muscles play an important role in providing proximal stability for distal mobility and limb functions during sporting activities (Kibler et al., 2006; Reed et al., 2012). In particular, the deep trunk muscles, including the transversus abdominis (TA) and multifidus muscle (MM), are involved in the stability of the trunk and are activated prior to the movement of the limbs (Hodges and Richardson, 1997a) in order to support the limb's power during motor chain activity (Butcher et al., 2007; Jamison et al., 2012). In addition, the psoas major muscle (PM) is suggested to stabilize the trunk as well as hip flexion (Santaguida and McGill, 1995). Therefore, deep trunk muscles provide a fundamental basis for the strength of the extremities, by acting in advance of other muscles, and thus affect sports performance.

Hibbs et al. (2008) suggested that an improvement of sports performance requires muscle hypertrophy in the trunk muscles. Only weak-to-moderate correlations were found between the core strength assessed by the endurance of the torso stabilizing muscles and sports performance (e.g., sprint run and jump; Nesser et al., 2008; Okada et al., 2011), which seems to negate the importance of core strength training for improving sports performance. However, this research assessed the endurance of the deep trunk muscles instead of hypertrophy. As such, it is necessary to evaluate morphological muscle hypertrophy in deep trunk muscles and determine their relationship with sports performance. The association of sprint performance with the size of the PM located in the deep part of the trunk has been previously reported (Hoshikawa et al., 2006; Ema et al., 2018), and most previous studies on sports performance and trunk muscles evaluated functional muscle strength and trunk stability (Nesser et al., 2008; Sharrock et al., 2011; Shinkle et al., 2012). Thus, the relationship between deep trunk muscle morphology and sports performance has not been investigated for other muscles. Based on the above findings, we hypothesized that the 100-m sprint performance correlates with the size of the deep trunk muscle.

Therefore, in this study, we aimed to explore the relationship between 100-m sprint performance and the cross-sectional area of PM and muscle thicknesses of the TA and MM associated with trunk stability in collegiate sprinters.

## Materials and Methods

### Participants

The participants of this study were 14 male sprinters who belonged to a university athletics club. Their age, height, and body weight (mean ± SD) were 20.1 ± 1.8 years, 172.0 ± 5.2 cm, and 65.6 ± 4.8 kg, respectively. Prior to the experiment, they were informed of the purpose, methods, and possible risks of this study. All participants provided written consent. This study was conducted with the approval of the Juntendo University Graduate School Ethics Committee (Approval number: #23-85).

### Muscle Thickness

Muscle thickness was measured using an ultrasonic diagnostic apparatus (ProSound C3; Aloka, Tokyo, Japan) using the B mode.

#### TA

**Figure 1:**
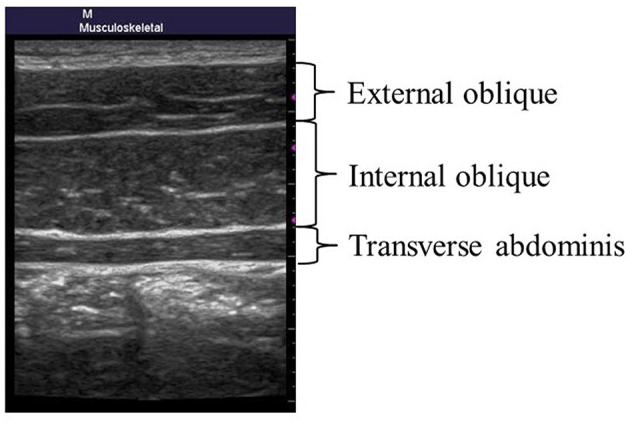
Measurement site of the transverse abdominis.

For the TA, participants lay on their backs and opened their lower limbs so that their thighs were parallel, with both shoulder joints at a 20–30 degrees transposition. The probe was placed at an inward position from the intersection point on the umbilical line and the underline of the lordosis (Hodges and Richardson, 1997b). The thickness (mm) of the TA was measured while confirming the image (Figure 1).

#### MM

**Figure 2:**
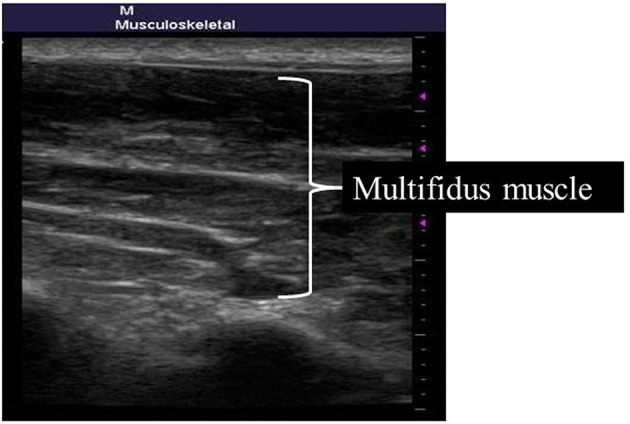
Measurement site of the multifidus muscle.

For the MM, participants lay in prone position and set the thighs parallel. The probe of the ultrasonic diagnostic device was placed on the side of the spinous process of the L4–L5 intervertebral joint, and the longitudinal image of MM was confirmed parallel to the spine (Hides et al., 1995). The thickness (cm) of the MM was measured while confirming the image (Figure 2).

### Cross-Sectional Area of the PM

A 1.5-T magnetic resonance imaging apparatus (Vantage Elan; Toshiba Medical Systems, Tokyo, Japan) was used for large psoas muscle cross-sectional imaging. T2-weighted images (FE method, TE: 90 ms, TR: 2,500 ms, matrix: 160 × 256, FOV: 320 × 32 mm, slice thickness 5 mm) of the central horizontal cross section between the fourth and fifth lumbar vertebrae were taken (Hoshikawa et al., 2006). The cross-sectional area (cm^2^) was calculated using image analysis software (OsiriX, 5.8.2, Pixmeo, Bernex, Switzerland). The sum of the left and right legs was used for the analysis.

### Statistical Analysis

All data are expressed as the mean ± SD, with the exception of 95% confidential interval (95% CI). For continuous variables, correlations were reported as Spearman product moment correlations. The statistical significance was set at *p* < 0.05. Mukaka's rule was used for the interpretation of the size of the correlation coefficient (Mukaka, 2012).

A change-point regression model (CPRM) was used to identify the optimal splitting point of the linear regression line (Hayamizu et al., 2009; Matsui et al., 2011). We estimated the point at which the time of the 100-m sprint became saturated using the CPRM. Akaike's information criterion (AIC) is a statistical value to express the goodness-of-fit in a model by imposing a penalty for increasing the number of parameters (Akaike, 1998). We used AIC to assess the goodness-of-fit in the models between the simple regression model and the CPRM for determining the presence/absence of the saturated points. Analyses were performed using R3.3.1 (R Core Team, 2018) and GraphPad Prism 6 software (GraphPad Software, San Diego, CA, USA).

## Results

The characteristics of the study participants are shown in Table 1. The cross-sectional area of the PM and thicknesses of TA and MM were 43.074 ± 7.35 cm^2^, 4.36 ± 0.72 mm, and 3.99 ± 0.48 cm, respectively. The mean 100-m sprint time was 11.00 ± 0.48 s. Spearman's correlation analysis found that 100-m sprint time had a significant moderate negative correlation with thickness of TA (ρ = −0.691, *p* < 0.01) and a low negative but not significant correlation with thickness of MM (ρ = −0.327, *p* = 0.28), whereas PM did not show a significant or in-negligible correlation (Table 2).

**Table 1:** Characteristics of the study participants.

			**95% CI**	
		**Mean ± SD**	**Lower**	**Upper**
Age	years	20.1 ± 1.9	19.0	21.2
Height	cm	172.0 ± 5.2	168.9	175.2
Body weight	kg	65.6 ± 4.8	62.7	68.5
100-m sprint time (season best)	s	11.0 ± 0.5	10.7	11.3
Psoas major (Cross-sectional area)	cm^2^	43.1 ± 7.4	38.6	47.5
Transversus abdominis (thickness)	mm	4.4 ± 0.7	3.9	4.8
Multifidus muscle (thickness)	cm	4.0 ± 0.5	3.7	4.3

**Table 2:** Spearman's correlation coefficients between 100-m sprint time and anthropometric parameters.

	**ρ**	***p***
Psoas major (cross-sectional area)	−0.226	0.46
Transversus abdominis (thickness)	−0.691	<0.01
Multifidus muscle (thickness)	−0.327	0.28

**Figure 3:**
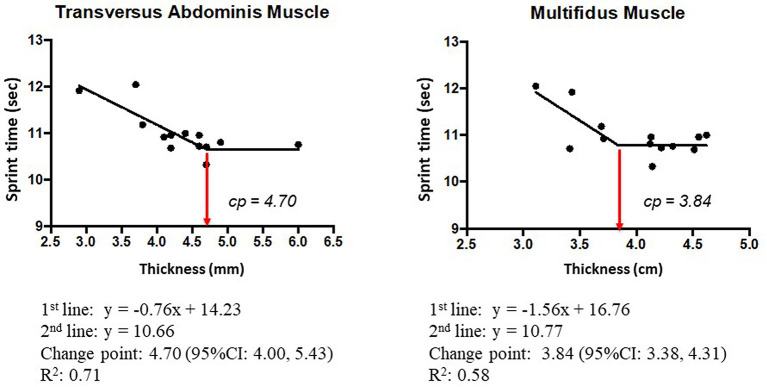
Mean profiles of the change-point regression model for the 100-m sprint time and the thickness of the TA and MM.

**Figure 4:**
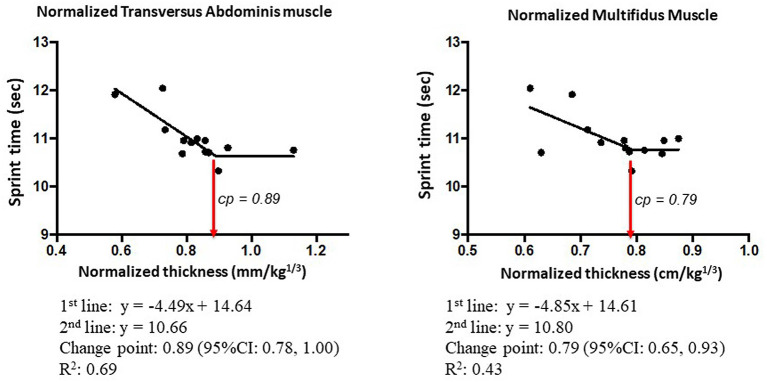
Mean profiles of the change-point regression model for the 100-m sprint time and the thickness of the TA and MM normalized by body mass.

The scatter plot of the 100-m sprint time and the thickness of the TA and MM depicted a biphasic relationship. Specifically, the sprint time decreased depending on the muscle thickness to a certain level, but the time became constant when the thickness became beyond a certain change-point. Therefore, we examined the CPRM for the presence of the change-points. The AICs of the CPRM were smaller than those of the simple regression model in both cases, which confirmed the presence of the change-points. The change-points of the TA and MM were estimated to be 4.70 mm (95% CI: 4.00–5.43 mm) and 3.84 cm (95% CI: 3.28–4.31 cm), respectively (Figure 3). The change-points were consistently observed when the thickness of the TA and MM was normalized by body mass: 0.98 mm/kg^1/3^ (95% CI: 0.78–1.00 mm/kg^1/3^) for TA and 0.79 cm/kg^1/3^ (95% CI: 0.65–0.93 cm/kg^1/3^) for MM (Figure 4). The stability of the change-points was confirmed by leave-one-out cross-validation, in which data sets were analyzed after excluding one participant's data for each analysis. As this study had 13 participants, we analyzed 13 data sets in the same manner for TA and MM with and without normalization by body mass.

In contrast, no change-point was observed between sprint time and cross-sectional area of PM with and without normalization by body mass (results not shown).

## Discussion

This study aimed to explore the relationship between the 100-m sprint time and the morphology of the deep trunk muscles in collegiate athletes. Sprint time exhibited a change-point at which the time decreased as the muscle thickness of the TA and MM increased, followed by a plateau phase; even if they became thicker. Thus, thickness of the trunk muscles did not show a simple linear correlation with the 100-m sprint time.

In this study, no correlation was found between the cross-sectional area of the PM and 100-m sprint time. Some studies have investigated junior athletes to demonstrate the association between the cross-sectional area of the PM and sprint performance (Hoshikawa et al., 2006; Tottori et al., 2018). Tottori et al. (2016) investigated sprinters and mid-distance runners of the same age as the present study; however, their sprint performance levels were lower compared with those of the participants of this study. In addition, the PM size of participants in this study (21.5 ± 3.7cm^2^) was larger than those measured by Hoshikawa et al. (2006) (17.1 ± 2.6 cm^2^) and Tottori et al. (2018) (8.6 ± 2.4 cm^2^). Kubo et al. (2011) reported that there was no relationship between sprint acceleration ability and cross-sectional area of PM, which is similar to the findings obtained in this study. Ema et al. (2018) demonstrated the importance of the PM, investigating the relation between actual running motion and muscle volume (not muscle size). Therefore, the muscle volume of PM instead of muscle size could be associated with sprint performance. Because there are few studies focusing on muscle volume, further research on sprint performance and muscle volume of PM should be conducted in adult sprinters with high sprint performance.

Previous studies have demonstrated the relationship between sprint performance and the extensor-flexor strength of the knee or hip (Alexander, 1989; Dowson et al., 1998). Hoshikawa et al. (2006) reported that 100-m sprint time was dependent on the PM-to-quadriceps femoris ratio rather than their absolute sizes. Another recent study found that the sizes of the thigh and PM, particularly the rectus femoris, may play an important role during the swing phase of sprinting (Ema et al., 2018). However, Spearman's correlation analysis showed that, instead of the PM, the deep trunk muscles, TA and MM had significant correlations with sprint performance. The TA and MM are classified as local muscles. The contraction of the TA enhances intra-abdominal pressure and thoracolumbar fascia tension (Cresswell et al., 1992). Elevated intra-abdominal pressure and thoracolumbar fascia tension work together to stabilize the spine (Gracovetsky et al., 1977). In addition, the contraction of the MM controls segments of the lumbar spine (Panjabi et al., 1989). Therefore, the TA and MM both might contribute toward stabilizing the spine and pelvis by controlling the intra-abdominal pressure (Cresswell et al., 1992), thoracolumbar fascia tension (Gracovetsky et al., 1977; Cresswell et al., 1992), and lumbar segments (Panjabi et al., 1989), and maintain the pelvis in an optimal position or posture for the running motion (Barr and Lewindon, 2014).

The activities of the muscles located from the shoulder to the pelvis have been shown to be important for transferring power from the larger torso to the smaller limbs (Stephenson and Swank, 2004). In addition, the 5,000 m running time of healthy adults was reported to improve with upper body training (Sato and Mokha, 2009). In contrast, some reports negate the importance of trunk training in sports performance (Nesser et al., 2008; Okada et al., 2011). However, previous studies have not investigated the thickness of deep trunk muscles. Here, we measured the muscle thickness of the deep trunk muscles to determine the importance of the trunk muscles in sprint performance.

Our findings demonstrated the existence of a change-point in the relationship between 100-m sprint time and the thickness of the TA and MM in both the actual thickness and thickness normalized by body mass. A possible reason for the change-point is the relationship between sprint time and physical load. In the sprint time up to 10.6 s, maintenance of trunk stability by the TA and MM contributes to performance; while in the time faster than 10.6 s, the increased physical load requires the contribution of other muscles in addition to TA and MM, e.g., abdominal oblique muscles and erector spinae. In fact, the anthropometric features of the 100-m finalists at the Olympics and World Championships apparently differ from those of the participants of this study. The mean height, body weight, body mass index, and 100-m sprint time were 176 ± 3.6 cm, 76.7 ± 6.4 kg, 25.5 ± 2.3 kg/m^2^, and 9.96 ± 0.5 s at the Beijing Olympics (2008); 177.3 ± 6.4 cm, 79.0 ± 8.0 kg, 22.5 ± 2.2 kg/m^2^, and 9.91 ± 0.10 s at the Berlin World Athletics Championships (2009); and 179.4 ± 8.1 cm, 80.4 ± 8.2 kg, 24.9 ± 1.5 kg/m^2^, and 9.86 ± 0.10 s at the London Olympics (2012) (Krzysztof and Mero, 2013). However, limited research makes it difficult to interpret this finding on local muscles and sprint performance. Accordingly, it is necessary to confirm whether the change-point can be observed in sprinter groups whose competition levels are higher/lower than the participants of this study.

The present study has some limitations. As we assessed the members of a university athletics club, the number of participants and diversity of their competition levels were limited. In order to minimize the impact of the small number of the participants, we performed leave-one-out cross-validation in CPRM and confirmed that stable results were obtained among the participants. However, further studies are warranted to validate the results. The extensor-flexor strengths of trunk muscles were not measured. The muscle strength can be estimated from muscle thickness (Muraki et al., 2013). However, in trunk muscles, the relationship between thickness and strength is not significant, suggesting that trunk muscle thickness may not directly affect muscle strength (Ishida et al., 2019). Therefore, further studies are warranted to determine the relationship of the TA and MM with sprint performance, considering anthropometric and muscle function (strength and power).

In this study, the local muscles, TA and MM, were shown to contribute to sprint performance for the first time. In addition, the existence of change-points indicates that simply aiming for the hypertrophy of these muscles would not be an effective strategy. These findings should provide important implications for sprint training.

## Data Availability Statement

All datasets generated for this study are included in the manuscript/supplementary files.

## Ethics Statement

The studies involving human participants were reviewed and approved by the Juntendo University Graduate School Ethics Committee. The patients/participants provided their written informed consent to participate in this study.

## Author Contributions

SF, SK, and KSakum: conceptualization. YSuz, YA, and KH: formal analysis. SF, SK, and AK: investigation. SF, AK, and KSakur: methodology. KSakum and KSakur: resources and supervision. SF, SK, YSug, and KH: writing–original draft. SF, YSuz, KH, and MS: writing–review and editing.

### Conflict of Interest

The authors declare that the research was conducted in the absence of any commercial or financial relationships that could be construed as a potential conflict of interest.
